# TOPSAN: use of a collaborative environment for annotating, analyzing and disseminating data on JCSG and PSI structures

**DOI:** 10.1107/S1744309110035736

**Published:** 2010-09-30

**Authors:** S. Sri Krishna, Dana Weekes, Constantina Bakolitsa, Marc-André Elsliger, Ian A. Wilson, Adam Godzik, John Wooley

**Affiliations:** a Joint Center for Structural Genomics, http://www.jcsg.org, USA; b Center for Research in Biological Systems, University of California, San Diego, La Jolla, California, USA; c Department of Molecular Biology, The Scripps Research Institute, La Jolla, California, USA; d Program on Bioinformatics and Systems Biology, Sanford–Burnham Medical Research Institute, La Jolla, California, USA

**Keywords:** collaborative annotations, structural genomics, Protein Structure Initiative

## Abstract

The NIH Protein Structure Initiative centers, such as the Joint Center for Structural Genomics (JCSG), have developed highly efficient technological platforms that are capable of experimentally determining the three-dimensional structures of hundreds of proteins per year. However, the overwhelming majority of the almost 5000 protein structures determined by these centers have yet to be described in the peer-reviewed literature. In a high-throughput structural genomics environment, the process of structure determination occurs independently of any associated experimental characterization of function, which creates a challenge for the annotation and analysis of structures and the publication of these results. This challenge has been addressed by developing TOPSAN (‘The Open Protein Structure Annotation Network’), which enables the generation of knowledge *via* collaborations among globally distributed contributors supported by automated amalgamation of available information. TOPSAN currently provides annotations for all protein structures determined by the JCSG in addition to preliminary annotations on a large number of structures from the other PSI production centers. TOPSAN-enabled collaborations have resulted in insightful structure–function analysis for many proteins and have led to numerous peer-reviewed publications, as exemplified by the articles included in this issue of *Acta Crystallographica Section F*.

## Abstract 2

Specific use cases of TOPSAN, an innovative collaborative platform for creating, sharing and distributing annotations and insights about protein structures, such as those determined by high-throughput structural genomics in the Protein Structure Initiative (PSI), are described. TOPSAN is the main annotation platform for JCSG structures and serves as a conduit for initiating collaborations with the biological community, as illustrated in this special issue of *Acta Crystallographica Section F*. Developed at the JCSG with the goal of opening a dialogue on the novel protein structures with the broader biological community, TOPSAN is a unique tool for fostering distributed collaborations and provides an efficient pathway to peer-reviewed publications.

## 1. Introduction

Our knowledge of the protein-structure universe has advanced significantly in the last decade owing in part to worldwide research efforts in high-throughput (HT) structural biology (Norvell & Berg, 2007 ▶). In the United States, such structural genomics efforts have been spearheaded by the Protein Structure Initiative (PSI; http://www.nigms.nih.gov/Initiatives/PSI/), which is funded by the National Institutes of Health (NIH) National Institute of General Medical Sciences (NIGMS; http://www.nigms.nih.gov/). The PSI centers have overcome numerous technical challenges and pioneered the development of HT pipelines for the determination of the three-dimensional structure of proteins, leading to date to close to 5000 protein structures. However, the analysis and communication of the biological insights revealed by these structures remains a major challenge. More details about the specific advances and contributions of our Center, the Joint Center for Structural Genomics (JCSG), are provided in the overview for the issue (Elsliger *et al.*, 2010 ▶).

Currently, the primary mechanism of sharing the results of the structures determined by the PSI centers is *via* deposition of the coordinates and experimental data in the Protein Data Bank (PDB) and public websites, such as the JCSG website at http://www.jcsg.org. The PSI–Structural Genomics KnowledgeBase (SGKB; http://kb.psi-structuralgenomics.org) provides a unified interface for the collection and collation of information from individual PSI centers and currently spearheads the effort in publicizing the achievements and successes of the PSI (Berman *et al.*, 2009 ▶). Structural genomics centers strive to publish the outcome of their efforts, doing so largely as brief peer-reviewed manuscripts such as the Structure Notes published by the journal *Proteins* (http://onlinelibrary.wiley.com/journal/10.1002/(ISSN)1097-0134/homepage/structure_notes.htm) or the Structural Comm­unications published by *Acta Crystallographica Section F*. These brief papers afford PSI scientists an opportunity to elaborate on the biological implications or potential functions that have been deduced from the protein structures to the broader scientific community. However, balancing precious resources to accommodate, on the one hand, production schedules and, on the other, research development, follow-up studies and publications has been a major challenge for the PSI centers. Thus, out of almost 5000 structures deposited in the PDB by PSI centers, only 20% have been described in peer-reviewed publications (http://olenka.med.virginia.edu/psi/). Owing to the significant increase in the production levels in PSI-2 by the four large-scale centers of around 200 structures a year, the percentage of structures solved in the last three years that have reached peer-reviewed publication is only 5–10%. Consequently, researchers who are pursuing studies of similar or related proteins may not even be aware of the availability and enormous utility of these structures for their own research.

To address this problem, the JCSG has pioneered the development of an open peer-reviewed communication platform to foster collab­orations to extend research on and annotation of the protein structures arising from structural genomics efforts. The Open Protein Structure Annotation Network (TOPSAN; http://www.topsan.org) was launched in 2006 as an extension of an internal system developed for effective management of protein-structure annotation and analysis efforts within the JCSG. TOPSAN embraces an open, community-wide, communication environment, but stresses authorship and quality control. TOPSAN provides a venue to present data and knowledge on targets and their structures, which gradually accumulate over time. As these entries mature, they can then be developed into traditional peer-reviewed papers such as those, for example, presented in this issue of *Acta Crystallographica Section F*. Furthermore, in the interim, invaluable information becomes publicly available to other groups pursuing synergistic research. In brief, TOPSAN serves as an incubator for the data emerging from discovery-driven science, promoting instant collaborations and providing connections to other relevant research.

## 2. Materials and methods

**Figure 1:**
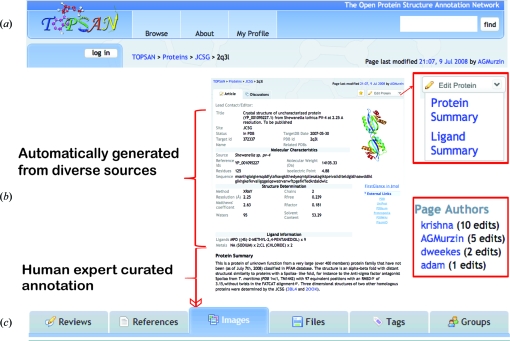
Features of the TOPSAN web portal. (*a*) The control panel allows TOPSAN users to securely login and contribute content. Registered participants can discover content by using various browse options or by searching for keywords. A version history that tracks all changes is stored on the wiki and is available for comparison to the current version of the article. (*b*) Registered users of TOPSAN participate in structure/function annotations of proteins by adding content to the protein and ligand summary sections and are credited for their individual contributions. Other parts of the protein page are generated automatically. (*c*) Additional features of TOPSAN include user comments and reviews, reference and figure management and keyword tagging of pages as well as the ability to group related protein pages automatically and/or *via* user input.

**Figure 2:**
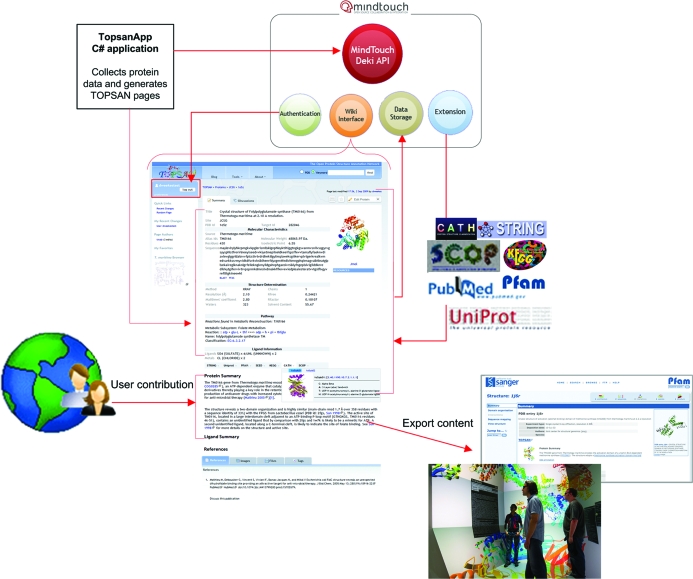
TOPSAN has been developed on the MindTouch Deki framework, which adheres to open standards and has a reasonable set of features that could be readily modified for our exploratory platform. A remote application TopsanApp uses the Deki’s API to generate and populate TOPSAN entries automatically with content from a variety of primary and derived data sources. Custom extensions allow real-time feeds from external data sources, which include CATH, SCOP, PubMed and UniProt, as well as SEED (Overbeek *et al.*, 2005 ▶), KEGG (Ogata *et al.*, 1999 ▶), PFAM (Sonnhammer *et al.*, 1997 ▶) and STRING (Snel *et al.*, 2000 ▶). The wiki interface allows community-based annotations, which can be exported through API access. UCSD’s CAVE display imports TOPSAN annotations in a structure-gallery wall display. PFAM 24.0 has integrated TOPSAN content on PFAM structure pages.

TOPSAN (Fig. 1 ▶; http://www.topsan.org) is built on top of the MindTouch Deki (http://www.mindtouch.com) collaboration platform *via* the addition of extensive scripting and modules that enable users to navigate through description pages, which integrate several sources and types of information including annotations that have been automatically parsed from established resources and user-created content and can be edited by any registered users (Weekes *et al.*, 2010 ▶). We developed a custom C# code built on the .NET framework to retrieve information from external resources to initial­ize and store TOPSAN entries for specified proteins through an Application Programming Interface (API). Initial TOPSAN entries are generated automatically by querying a series of internal (in the case of JCSG targets) and external, well established data sources (see Fig. 2 ▶) to retrieve information specific to each protein. All collected data are stored in a local MySQL database, topsanDB, which is used to generate the TOPSAN pages *via* Deki’s REST API (Weekes *et al.*, 2010 ▶). Each TOPSAN page contains a Dekiscript query retrieving target data from topsanDB, which in turn are passed as arguments to a Deki template. This approach provides easy content management and uniformity across the site (Fig. 2 ▶). Viewing or editing of pages can be controlled either at the individual user or group level through tiered access control. Only registered users are permitted to modify content and these contributions are subject to editorial review. However, the entire content of TOPSAN is open access and can be viewed by all users of the site and indexed by search engines, such as Google and Bing.

The process of annotation and analysis of each protein can typically be divided into two phases. The first involves gathering standardized data, such as amino-acid sequence and molecular weight from the PDB and structural or functional classification from SCOP (Murzin *et al.*, 1995 ▶), CATH (Orengo *et al.*, 1997 ▶) and UniProt (Apweiler *et al.*, 2004 ▶). This process is mostly carried out by automated scripts during the initial creation of the page and can be revised upon request. The analysis phase, which builds on the first information-gathering phase, involves analysis and interpretation of the available data together with the results of any additional focused experiments. This second phase cannot be meaningfully automated and, thus, is manually curated by experienced researchers in the field. The TOPSAN platform (Fig. 1 ▶) greatly facilitates all stages of the process. Data automatically pulled from external databases and/or pre-computed by annotation scripts are inserted into a template with standardized fields for user input. Navigation and editing of the resulting page is facilitated *via* a versatile WYSIWYG editor. User-provided annotations can include figures in addition to text and bibliographical links.

TOPSAN aims not only to collect information, but also to create and disseminate knowledge. TOPSAN pages are free to view without registration and search engines such as Bing and Google, have now become the point of origin for most new viewers of TOPSAN pages. TOPSAN content is licensed under the Creative Commons share-alike license (http://creativecommons.org/licenses/by-nc-sa/3.0/legalcode), which allows others to legally build upon and share it. In addition to open-content licensing, the TOPSAN API enables external sources to easily retrieve content and a custom PHP script (http://files.topsan.org/retrieve.php?uniprotId=xxx) was developed for servers to readily access TOPSAN page content in real time. When a page is requested, the script queries the TOPSAN MySQL database for the current annotation of the page as well as any file attachments/images. This information is formatted as an XML document and returned to the client. This method of retrieving information from TOPSAN is used, for instance, by the Calit2 Visualization Lab at UCSD, which displays TOPSAN protein annotations within their structure gallery CAVE 3D wall display (Fig. 2 ▶), and by PFAM, which starting from release 24.0 shows real-time feeds of TOPSAN pages on its site (http://pfam.sanger.ac.uk). 

## 3. Results and discussion

**Figure 3:**
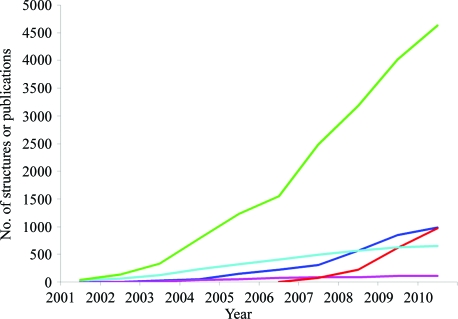
Useful annotations of nearly all of the structures solved by the JCSG have been generated and shared with the public by use of the community-based collaboration platform TOPSAN, whereas traditional methods of publication provide information for only a fraction of the JCSG structures. Dark blue, JCSG structures deposited in the PDB. Purple, JCSG structure publications. Red, structures in TOPSAN. Light blue, PSI structure publications. Green, PSI structures deposited in the PDB.

Structural genomics centers originally envisioned using the traditional publication model to disseminate the insights extracted from the structures that they determined. However, the number of publications, including both brief structure notes (*i.e.* Structure Communi­cations) and full-sized articles, cannot keep pace with the current speed of structure determination. For example, the JCSG has published over 100 peer-reviewed structural papers compared with the more than 1000 structures that it has determined and deposited in the PDB (Fig. 3 ▶). In contrast, TOPSAN, as of May 2010, provides entries containing curated analyses for all of the structures determined by the JCSG. Additionally, TOPSAN contains entries with analysis for many proteins in the production pipeline that have no associated structure, yet were of interest, for various reasons, to TOPSAN users. These contributions vary in length, ranging from a single paragraph to several pages, with a median of about 150 words, and in quality, from relatively trivial annotations to those of publication quality (Fig. 3 ▶). In addition, TOPSAN has entries for the structures determined at other structural genomics centers and over 4700 entries for JCSG targets that are at various stages of production through our pipeline.

TOPSAN offers several automated and user-operable tools that can help accelerate the exploitation of structural genomics structures and support a pathway to publication. Links to external resources [*e.g.* PDBsum (Laskowski *et al.*, 1997 ▶) and ProFunc (Laskowski *et al.*, 2005 ▶)] allow users to update database searches and comparisons, aggregate information and enable meta-analysis. Content evaluation is offered, initially through in-house curators and additionally by invited outside experts. Such multi-directional information transfer has been very powerful in enriching TOPSAN content, providing both context and integration. This can be further enhanced by user-defined groupings and tags that group proteins at a higher level of classification, such as folds, protein families and specific pathways of organisms.

Currently, TOPSAN contains close to 300 ‘protein groups’, mostly for proteins of unknown function (DUF) solved at PSI centers, and two ‘organism pages’ for JCSG target organisms: the hyperthermophilic bacterium *Thermatoga maritima* (Kuhn *et al.*, 2002 ▶; Zhang *et al.*, 2009 ▶) and the dominant human gut symbiont *Bacillus thetaiota­omicron*. Users can monitor specific pages and content for any changes made by others *via* RSS feeds (star icon, envelope icon). Authorship tracking is provided *via* time-stamped archives that ensure assignment of credit for all contributions. Future development will allow users to attach specific tools which are tailored to their area of research and expertise.

Since all information on TOPSAN is public, the issues of data ownership that typically limit access to unpublished data do not impede content analyses by third-party participants. The collaborative process used to achieve understanding is itself archived on TOPSAN. Once the discussion has matured (*i.e.* is both deep in content and has achieved convergence in point of view), TOPSAN serves as a conduit to peer-reviewed publication. In turn, having these insights and discussions posted in TOPSAN ensures that the content of the TOPSAN pages attains conventional validation. We describe below three examples of TOPSAN-enabled scientific interactions or dialogues among distributed participants (who were initially un­known to each other) that led to significant functional insights in the analysis of specific JCSG structures. These interactions have led to manuscripts in three different sections of this special issue; a detailed history of these interactions provides different scenarios on the range of usage of the TOPSAN system. Many of the articles included in this special issue of *Acta Crystallographica Section F* also involved key input from such TOPSAN-enabled discussions.

### 3.1. Example 1. DUF1470: novel classification (structure, family) and data integration

The structure of the DUF1470-family member Jann_2411 (Bakolitsa *et al.*, 2010 ▶) unexpectedly revealed a two-domain organization rather than the single domain that was previously described in the Pfam classification. This finding prompted re-evaluation of this Pfam family by Dr Alex Bateman, which resulted in this family being split into two new entries, the first comprising the N-terminal domain (a new fold that is now named the ABATE domain for Alpha-Beta-hairpin-Alpha TandEm) and the second covering the C-terminal treble-clef zinc-finger domain (PDB code 3h0n). A combination of genomic context and sequence-conservation pattern analysis in addition to literature searches allowed data integration and the placement of DUF1470 in a biological context, suggesting that this family is likely to be involved in DNA binding with a role in stress-induced transcriptional regulation (Bakolitsa *et al.*, 2010 ▶).

### 3.2. Example 2. DUF35: novel classification (family, function–domain combination) and data integration

The structure of the DUF35-family member SSO2064 from *Sulfolobus solfataricus* determined by the JCSG revealed a two-domain architecture comprising an N-terminal zinc-ribbon domain and a C-terminal oligonucleotide/oligosaccharide-binding fold (OB-fold) domain (PDB code 3irb; Krishna *et al.*, 2010 ▶). Gene fusion, genomic context analysis and functional evidence from certain bacterial representatives of this family suggest that these proteins form a novel component of lipid and polyketide antibiotic bio­synthesis and possibly function as acyl-CoA-binding proteins (Krishna *et al.*, 2010 ▶). The domain delineation and structural analysis were carried out by Dr Sri Krishna from the JCSG and the genomic context/subject expertise was provided by Dr Aravind from the National Center for Biotechnology Information (NCBI). This collaborative study led to the prediction that DUF35 members are a novel group of proteins in which the interface between the OB-fold and zinc-ribbon domains has been adapted to bind an acyl-CoA moiety. The structure also led to a re-evaluation of the DUF35 family, which has been split into two entries in the most recent release on protein families (Pfam 24.0).

### 3.3. Example 3. DUF3478: novel classifications (structure, family), association and data integration

The structure of two proteins YP_749275.1 and YP_001095227.1 from *Shewanella* spp. from the newly created Pfam family DUF3478 (assigned by Dr Bateman based on JCSG structures) adopts an α+β fold with a distant structural similarity to proteins with a SpoIIAA-like fold. Both proteins (PDB codes 2ook and 2q3l) are apparent orthologs of the possible universal stress protein SO3682 from *S. oneidensis* MR-1. Dr Alexey Murzin indicated *via* TOPSAN (http://www.topsan.org/explore?PDBid=2ook) that despite a very high sequence identity (54%) the two structures reveal large-scale differences in subunit conformations and brought to our attention his recent paper (Murzin, 2008 ▶) on metamorphic proteins in which he presented his analysis of these structures.

Independently, Dr Andrei Lomize, a scientist who develops software for fold recognition and modeling of membrane proteins, was interested in members of this protein family. Dr Lomize had previously contributed to TOPSAN in the annotation of another membrane-interacting protein that is also part of this special issue (Bakolitsa *et al.*, 2010 ▶). Subsequently, he contacted Dr Bakolitsa, his previous contact at JCSG, about the DUF3478 structures, as he believed they were very strong candidates for membrane interactions. The collaboration that ensued allowed different experts to express and discuss their views and eventually led to a publication (Kumar *et al.*, 2010 ▶) that predicted the function of these two proteins as possible water-soluble carriers of hydrophobic compounds.

## 4. Conclusions

TOPSAN offers a solution for the challenges inherent in HT structure determination, which could also be relevant to more general challenges of effective scientific communication in the era of high-throughput technology-driven biological science. Furthermore, the TOPSAN approach could be invaluable to structural biologists in general as a means to communicate more effectively with the bio­logical community and provide a forum for discussion and ideas, as well as accumulation of data and knowledge, on biological macromolecules and systems prior to and even post publication. On a first level, TOPSAN provides a means for more rapid dissemination of structural results from structural genomics from the JCSG and other SG efforts. On a second level, TOPSAN also serves as a model system for engaging the broader scientific community by contributing to the understanding and functional annotation of structural genomics data. This type of engagement is particularly important for structural genomics, where the rapid pace of structure determination means that the centers themselves do not have the resources or the specific expertise to provide rich annotation for each structure determined and automated analyses cannot replace expert manual analysis of protein structures. TOPSAN also reflects the potential of ‘Open Lab-Book’ or ‘Early Access to Experimental Data’, which is among the communication processes being explored in the biological community (http://ccbw.calit2.net/). Through providing direct access to annotation on TOPSAN introduced as part of the JCSG efforts, we in essence invite the community to participate in our experiments.

Maximizing access to available information and expertise is necessary for full understanding of the implications of the novel protein structures that are being solved in the high-throughput mode of structural genomics. Many of the targets are defined as proteins of unknown function and are intentionally chosen for that reason by the PSI structural genomics centers. The goal then is to provide a mechanism to annotate and define their function. This fascinating unexplored sector of the protein universe has been expanding owing to the ever-increasing numbers of complete genomes that are being sequenced. By capturing, organizing, communicating and sharing the information delivered through this focused large-scale effort on protein structures that represent this expanding protein universe, as well as the more focused efforts on important biological systems, TOPSAN provides a powerful tool to facilitate the awareness, access and usage of these structures by the biological community. In this way, the unique contributions of the PSI can be better harnessed to accelerate the discovery of the biological roles and functions of the proteins whose structures have been determined.
